# Two new genera of big-eyed minute litter bugs (Hemiptera, Schizopteridae, Hypselosomatinae) from Brazil and the Caribbean

**DOI:** 10.3897/zookeys.640.9690

**Published:** 2016-12-13

**Authors:** Rochelle Hoey-Chamberlain, Christiane Weirauch

**Affiliations:** 1 Department of Entomology, University of California, Riverside, 900 University Ave., Riverside, CA 92521, USA

**Keywords:** Dipsocoromorpha, taxonomy, morphology, Neotropics, Hypselosomops pecki, Hypsohapsis takiyae

## Abstract

Charismatic Hypselosomatinae (currently 14 extant and fossil genera; 72 species), the “big-eyed minute litter bugs”, are characterized among Schizopteridae (Dipsocoromorpha) by the large eyes, four-segmented labium, and distinctive wing venation. A recent molecular phylogenetic analysis confirmed the monophyly of Hypselosomatinae that were recovered as the sister taxon to the Ogeriinae + Schizopterinae (Weirauch and Štys 2014). Hypselosomatinae occur in the Old and New Worlds, but described species diversity is biased towards the Oriental and Australian regions: only three monotypic genera are currently known from the New World (*Glyptocombus* Heidemann, *Ommatides* Uhler, and *Williamsocoris* Carpintero & Dellapé). Based on 28 male, female, and juvenile specimens from Cuba and the Dominican Republic and a single male specimen from Brazil we here describe two new monotypic genera of Hypselosomatinae, *Hypselosomops pecki* **gen. n. and sp. n.**, and *Hypsohapsis takiyae* **gen. n. and sp. n.** We provide habitus images, digital illustrations (light, scanning electron, and/or confocal microscope) of wing and male genitalic structures, line drawings of genitalic structures, and distribution maps. Bizarre morphologies, a worldwide distribution with small endemic species ranges, and a fossil record that dates back to the mid-Cretaceous make the Hypselosomatinae a fascinating group to explore in an effort to understand the evolutionary history of Dipsocoromorpha.

## Introduction

Schizopteridae are tiny (1–3 mm) yet charismatic Heteroptera, the study of which is hampered by their cryptic habits and small size that make them rare in collections and difficult to examine. Adults display extreme wing polymorphism ranging from apterous to macropterous and elytrous, with the latter being a wing type that is very rare outside of Coleoptera. Wing polymorphism can be sexually dimorphic (Wygodzinsky 1948, Emsley 1969, Hill 2013, Knyshov et al. 2016, Leon and Weirauch 2016), but may also occur amongst individuals of the same sex (Hill 1980; Alexander Knyshov, pers. obs.). In addition, extreme asymmetry of the male genitalia, a feature that is fairly rare in Heteroptera, is widespread in Schizopteridae and often includes modifications of the pre-genital abdomen (Emsley 1969, Štys 1970). With 72 described species, the subfamily Hypselosomatinae, also known as “big-eyed minute litter bugs”, is currently the second largest subfamily of Schizopteridae. Diagnostic features include the very large eyes, often overlapping the margins of the prothorax, four-segmented labium, well developed ovipositor, and distinctive wing venation of four closed marginal cells after the costal cell (Emsley 1969, Lionel Hill 1980). Hill (1980) in addition discussed the following characters as potentially diagnostic for the subfamily: anterior sclerites formed from the ninth abdominal segment and forming part of the ovipositor (referred to by Hill (1980) as gonangulum struts) fused to ninth segment; three, six or seven pairs of abdominal spiracles; hind wing, when present, with jugal lobe; and possession of gonoplacs in females. Emsley (1969) recognized Hypselosomatinae as a well-defined group in his schema of proposed relationships among Schizopteridae, and the only published phylogenetic analysis confirmed the monophyly of this group with high branch support, although only four of the currently recognized 14 genera were represented in that analysis (Weirauch and Štys 2014).

The taxonomic history of this group started in the late 19^th^ century with the almost simultaneous description of Old World *Hypselosoma oculata* Reuter by Reuter (1894) and New World *Ommatides insignis* Uhler by Uhler (1894). A second New World genus, *Glyptocombus* Heidemann, was described after the turn of the century (Heidemann 1906) and various scientists added species to the Old World genus *Hypselosoma* Reuter: Horvath (1905) [1 species], Esaki and Miyamoto (1959) [1 species], McAtee and Malloch (1925) [1 species], and Wygodzinsky (1960) [9 species]. Much later, the hypselosomatine diversity of Australia and adjacent regions was extensively studied by the most prolific taxonomist to focus on this subfamily, Hill, who has described 7 new genera and 53 new species during the past 25 years (*Pateena* Hill, *Macromannus* Hill, *Ordirete* Hill, *Lativena* Hill, *Cryptomannus* Hill, *Duonota* Hill, *Rectilamina* Hill (Hill 1980, 1984, 1985a, 1985b, 1987, 1991). The third New World genus, *Williamsocoris* Carpintero and Dellapé, was recently described from Argentina (Carpintero and Dellapé 2006). Together with three monotypic genera that are based on fossils, *Libanohypselosoma* Azar and Nel, *Buzinia* Perrichot, Nel and Néraudeau, and *Tanaia* Perrichot, Nel and Néraudeau (Azar and Nel 2010, Perrichot et al. 2007), 72 species in 14 genera are currently recognized in the Hypselosomatinae.

**Figure 1.**
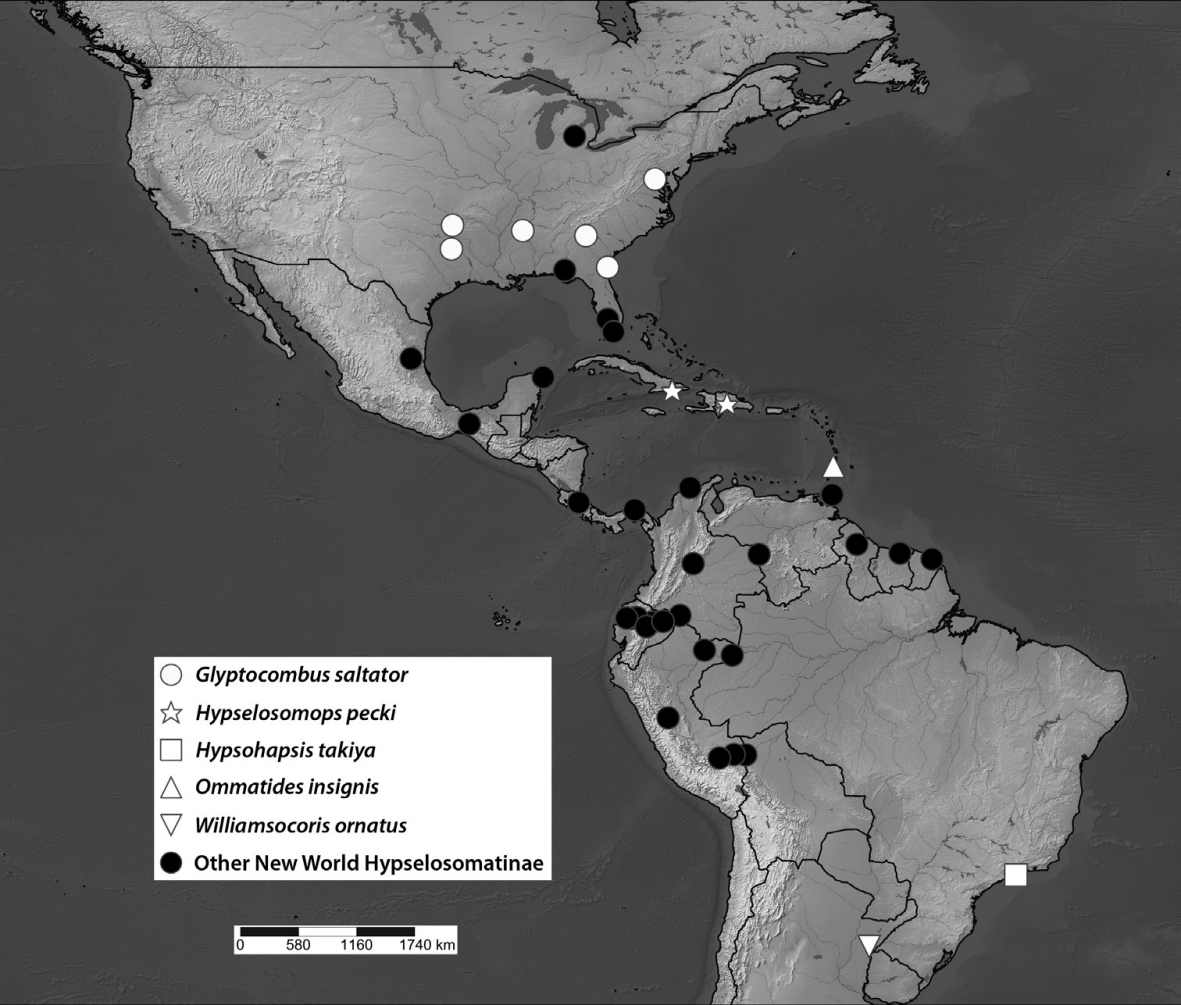
Map of distribution of all New World Hypselosomatinae (generated using data entered in the American Museum of Natural History’s Arthropod Easy Capture Software database (http://www.research.amnh.org/pbi/locality/index.php))

The Hypselosomatinae have a worldwide distribution but are most diverse in the tropics, a pattern similar to those observed in other groups of Dipsocoromorpha. The greatest described diversity is from the Indo–Pacific and Australia, but this may be an artifact due to Hill’s focus on that biogeographic region and the lack of research on the Oriental, Afrotropical, and Neotropical regions. Hypselosomatines inhabit cryptic and typically moist microhabitats such as leaf litter, low vegetation, the interstitial zone of streams, and even mangroves (McAtee and Malloch 1925, Emsley 1969, Hill 1984, 1991, Melber and Köhler 1992, Schuh and Slater 1995, Ng et al. 1999). Published distributional data are scarce for Hypselosomatinae beyond the well-documented Indo–Pacific and Australian fauna. Despite the fact that Hill (1984) suspected that new taxa of hypselosomatines are unlikely to be discovered in South America, the recently described *Williamsocoris* was collected in Argentina. Along similar lines, Wygodzinsky (1960) commented on the absence of Hypselosomatinae in Africa despite their presence in Madagascar. Based on examination of schizopterid specimens that we have assembled from various natural history collections and that include ~632 specimens of Hypselosomatinae, we have discovered a number of undescribed Hypselosomatinae from Central and South America, Africa, and the Indo–Pacific region. In the New World, we have examined Hypselosomatinae from Panama, Suriname, Ecuador, Peru, Dominican Republic, Costa Rica, Mexico, USA [FL, GA, TX, OK, AR, KS, TN, MD, MI], Trinidad and Tobago, French Guiana, Colombia, Cuba, and Brazil (Fig. 1). This publication focuses on a subset of these specimens, specifically specimens from Cuba, the Dominican Republic, and Brazil that represent two new monotypic genera that we here describe and document using light, scanning electron, and confocal microscopy. Future publications will focus on the remaining specimens from other localities that are likely new species of already described genera.

## Materials and methods

### Material

A single male specimen collected in Sao Paolo, Brazil (BRAZIL: Sao Paulo: Ubatuba: Picinguata; -23.37743, -44.83733; 1997–2001, Nessimain, J.L. & Takiya, D.M.), loaned to us by Daniela Takiya, will be deposited in the Universidade Federal do Rio de Janeiro (UFRJ). Habitus images were taken and the specimen was then DNA extracted using a non-destructive protocol and Qiagen DNeasy Blood and Tissue Kit (Qiagen, Hilden, Germany) in a solution of ATL buffer and protease K for later sequencing. This resulted in slight clearing of the specimen which allowed further dissection. The abdomen of the specimen was removed and temporarily slide mounted in glycerin gelatin for examination and documentation through images and drawings. The entire specimen was then permanently mounted on a microscope slide using Canada balsam. In addition, 28 specimens (14 adult male, 8 adult female, and 7 juveniles) collected in Cuba and the Dominican Republic loaned to us by the Field Museum of Natural History in Chicago, IL, USA were examined. Seven specimens were imaged for habitus photo plates. The three adult specimens (UCR_ENT 00091115, UCR_ENT 00091116, and UCR_ENT 00091117; see below for explanation of specimen codes) were DNA extracted following the above described protocol. Four specimens were dissected by removing the abdomen, leaving the abdomen intact for males, but removing soft tissue using KOH clearing for females (UCR_ENT 00091115, UCR_ENT 00091117, UCR_ENT 00091118, UCR_ENT 00096904–UCR_ENT 0009607). Two specimens (one male and one female) were dissected in preparation for scanning electron microscopy (UCR_ENT 00096908 and UCR_ENT 00098865).

### Imaging, dissections, measurements, databasing and distribution maps

Dorsal, lateral, and ventral habitus images were produced using a Leica DFC 450 C Microsystems system (Leica, Wetzlar, Germany) with a Planapo 1.0x objective. Images of select morphological features were produced using a Leica DFC 450 C Microsystems system (Leica, Wetzlar, Germany) with a Planapo 2.0x objective. Images were stacked using the Leica Application Suite V4.3 software or Zerene stacker V1.02 (Zerene Systems). Confocal images of genitalia were taken on a Leica SP5 Inverted confocal microscope at the Institute for Integrative Genome Biology core facility (http://genomics.ucr.edu/) using cuticular auto fluorescence excited by 488nm and 543nm lasers and collected by detectors in diapasons of 500–535nm (green in figures) and 555–700nm (red in figures). The resulting confocal images were rendered using Imaris V7.6.4 (Bitplane). Specimens used for Scanning Electron Microscopy were sputter coated with a platinum-paladium using a Cressington 108 auto sputter coater (Cressington, Watford, UK) before imaging using a FEI XL30-FEG Scanning Electron Microscope at the UCR Central Facility for Advanced Microscopy and Microanalysis (http://cfamm.ucr.edu/). All images were edited and assembled into image plates in Adobe Photoshop CS4.

To examine male genitalia, the abdomen was separated from the body and either cleared by placing into hot 10% KOH, or prepared for DNA extraction. For study of female genitalia the abdomen was separated from the body, cleared in KOH for ~10 minutes, and placed in Chlorazol Black E for three 30 second intervals. Line drawings were prepared using a Nikon Eclipse 80i compound microscope (Nikon, Tokio, Japan) with camera lucida.

Measurements (see Table 1) were made using habitus images and all measurements are in mm. Total length was measured from the front of head to apex of forewing, body length was measured from the front of head to apex of abdomen, and width between eyes was measured in frontal perspective.

**Table 1.** Specimen measurements in mm.

Species	*Hypselosomops pecki*							*Hypsohapsis takiyae*
Sex	Male (macropterous)	Male (brachypterous)	Female	1st instar	2nd instar	4th instar	5th instar	Male
**USI**	UCR_ENT 00091116	UCR_ENT 00091117	UCR_ENT 00096912	UCR_ENT 00096916	UCR_ENT 00096917	UCR_ENT 00096918	UCR_ENT 00096919	UCR_ENT 00111639
**Total length**	1.49	1.03	1.09					1.51
**Body length**	1.53	1.03	1.38	0.69	0.80	1.03	1.13	1.48
**Pronotum width**	0.61	0.53	0.54					0.74
**Pronotum length**	0.16	0.23	0.18					0.26
**Pronotal collar length**	0.04	0.07	0.05					0.05
**LC:LP**	0.23	0.28	0.28					0.21
**Vertex width**	0.36	0.36	0.37	0.28	0.22	0.31	0.33	0.50
**Eye width**	0.68	0.65	0.67	0.41	0.46	0.60	0.61	0.92
**Width between eyes**								0.37
**Fore femora depth/ height**	0.07	0.08	0.08	0.05	0.05	0.07	0.07	0.17
**Fore femora length**	0.28	0.30	0.28	0.17	0.13	0.24	0.25	0.56
**DFF:LFF**	0.25	0.26	0.29	0.30	0.41	0.31	0.30	0.30
**Hind tibia length**	0.61	0.59	0.56	0.24	0.24	0.30	0.42	0.72
**LHT:WP**	1.00	1.11	1.03					0.97
**a1 length**		0.08						
**a2 length**		0.07						
**a3 length**		0.28						
**a4 length**		0.30						
**a3:a4**		0.93						


 Unique specimen identifier (USI) labels consisting of a matrix-code and prefix plus eight-digit number were associated with each specimen. Specimen information was databased using the American Museum of Natural History’s Arthropod Easy Capture (AEC) Software database (http://www.research.amnh.org/pbi/locality/index.php). Specimens for which geographic coordinates were absent from the label were georeferenced using Google Earth. The associated information is available through the Heteroptera Species Pages (http://research.amnh.org/pbi/heteropteraspeciespage/) and the http://www.discoverlife.org/ website. Specimens stored in ethanol and voucher specimens for molecular study were also given an internal identification number. Maps were prepared in SimpleMappr (http://www.simplemappr.net/) using the coordinates exported from the AEC database.

### Nomenclatural acts

This publication and the nomenclatural acts it contains have been registered in ZooBank, the online registration system for the ICZN. The ZooBank LSIDs can be resolved by appending them to the Web address http://zoobank.org/. The LSIDs for nomenclatural acts can be found in corresponding sections of this article.

### Terminology and abbreviations (for tables and figures)

We use the following abbreviations: 1An, first anal vein; 2An, second anal vein; ac, anal cell; ano, anophore; anop, anophoric process; ag, anterior gonapophysis; at, anal tube; bucc, buccula; ca, conjunctival appendage of vesica; cly, clypeus; clypD, clypeal depression; C, costa; cc, costal cell; Cu, cubitus; cub, cubital cell; dc, discal cell; DFF:LFF, ratio of height (termed depth in Hill (1980)) of fore femora to length of fore femora; g, gonoplacs; ipt8, internal process of tergite eight; l1, first labial segment; l2, second labial segment; l3, third labial segment; l4, fourth labial segment; labr, labrum; LC:LP, ratio of pronotal collar length to pronotum length; LHT:WP, ratio of length of hind tibia to width of pronotum; lp, left paramere; M, media; M1, first branch of media; M2, second branch of media; mdp, mandibular plate; flp, flap like process; mxp, maxillary plate; pg, posterior gonapophysis; R, radius; R1, first branch of radius; R2, second branch of radius; rp, right paramere; s7, sternite seven; s8, sternite eight; s9, sternite nine; Sc, subcosta; spd, spermathecal duct; spgd, spermathecal gland duct; spgl, spermathecal gland; spr, spermathecal reservoir; spth, spermatheca; st, styloid; t1–2, tergite one and two; t3, 4, 5, 6, 7, tergites three, four, five, six, seven; t8, tergite eight; t8p1, anterior most process of tergite eight process; t8p2, posterior most process tergite eight; tc, trapezoidal cell; v, vesica; wc, wing coupling organ.

Hill (2013) used terms including terminal capsule, apical canal, globular body, and duct to describe parts of the female spermatheca. Instead, we here use the terms spermathecal gland, spermathecal gland duct, spermathecal reservoir, and spermathecal duct that are both more descriptive and more widely used. The structure referred to as clypeus by Hill (1984) but later corrected in Hill (2014) and reiterated in Hill (2015) is the labrum and we refer to it accordingly. Hill (1984) used the term postnotum which he defines as a posteriorly “directed flange of the metanotum occupying up to half of the width of the pterothorax at the point where it project freely as a flange,” we will use this term as well.

### Identification Key

Key to the genera of Hypselosomatinae in the Old and New Worlds (modified from Hill 1984), indicating known distribution of genus (NW, New World; OW, Old World)

**Table:** 

1	First labial segment dorsally expanded, appearing lobelike in lateral view (Fig. 12B & C)	**2**
–	First labial segment not dorsally expanded	**4**
2	Third and fourth labial segments not distinctly separated, with a sclerotized, hornlike or tubular structure occupying both segments (Fig. 12D)	***Williamsocoris* Carpintero & Dellapé** (NW)
–	Third and fourth labial segments distinctly separated and without any ornamentation	**3**
3	Males with slightly enlarged first labial segment lacking anterior teethlike setae; clypeus of male lacking macrosetae and medially depressed with posterior flaplike expansion (Fig. 5C)	***Hypselosomops* gen. n.** (NW)
–	First labial segment significantly enlarged, containing anterior teethlike setae (Fig. 12A & B); clypeus convex with three macrosetae (Fig. 12 bottom left)	***Ommatides* Uhler** (NW)
4	Clypeus without macrosetae	**5**
–	Clypeus with 1–5 macrosetae; female never macropterous	**6**
5	Macropterous	major part of ***Rectilamina* Hill** (OW)
–	Elytrous (Fig. 12)	***Glyptocombus* Heidemann** (NW)
6	Clypeus with 1 macroseta	7
–	Clypeus with 3–5 macrosetae	**8**
7	Vertex deflected 90° at posterior margin, with median furrow; venation normal width	***Cryptomannus* Hill** (OW)
–	Vertex not sharply deflected, without furrow; elytrous venation very wide	***Lativena* Hill** (OW)
8	Clypeus with 5 macrosetae	**9**
–	Clypeus with 3 macrosetae	**10**
9	Fore femur with long macrosetae	***Hypselosoma* Wygodzinsky** (OW)
–	Fore femur without macrosetae (Fig. 8)	***Hypsohapsis* gen. n.** (NW)
10	Ratio of pronotal collar length to pronotum length ≥ 0.29 (elytrous), 0.25 (macropterous); Ratio of length of hind tibia to width of pronotum < 0.95	**11**
–	Ratio of pronotal collar length to pronotum length ≤ 0.25; ratio of length of hind tibia to width of pronotum > 0.95	**12**
11	Body black	***Pateena* Hill** (OW)
–	Body brown	***Macromannus* Hill** (OW)
12	Pronotal disc with transverse furrow; elytrous forewing cells with regular rows of large ovoid punctures	***Ordirete* Hill** (OW)
–	Disc with no furrow; cells not coarsely sculptured	**13**
13	First antennal segment with short macrosetae; second hind tarsomere with 2 short to medium macrosetae	major part of ***Duonota* Hill** (OW)
–	First antennal segment without macrosetae; second hind tarsomere with 1 medium macrosetae (ignore long apical ventral seta)	**14**
14	Male genital capsule tapering to single curved process; proepisternum and pronotum black-brown to black	***Rectilamina curvicauda* Hill** (OW)
–	Capsule projecting at both posterior corners; proepisternum cinnamon, pronotum black with posterior angles cinnamon	***Duonota bicamaca* Hill** (OW)
–	Pronotal disc with 2 eye-like yellow spots, proepisternum brown; male unknown	***Duonota bimaculata* Hill** (OW)

## Taxonomy

### Hypselosomops gen. n.

Taxon classificationAnimaliaHemipteraSchizopteridae

http://zoobank.org/364FFCA8-2B30-4F67-A09E-E17258ACFDBE

**Figure 2.**
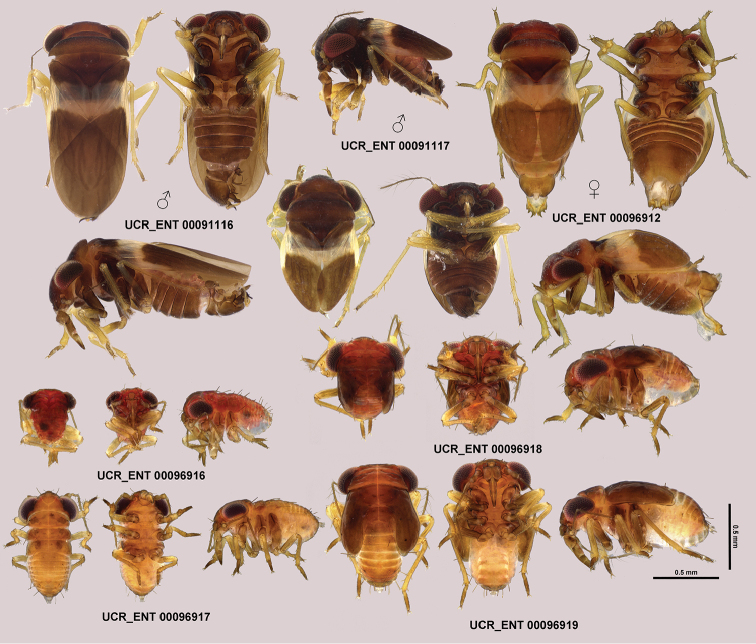
Dorsal, ventral, and lateral habitus of macropterous and submacropterous males, female, and first, second, fourth, and fifth instar juveniles of *Hypselosomops pecki*.

**Figure 3.**
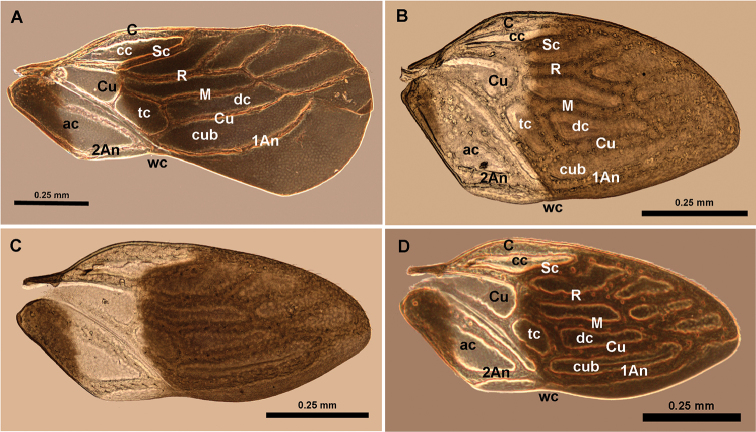
**A** Macropterous male wing **B** submacropterous female wing: **C, D** submacropterous male wing of *Hypselosomops pecki*.

**Figure 4.**
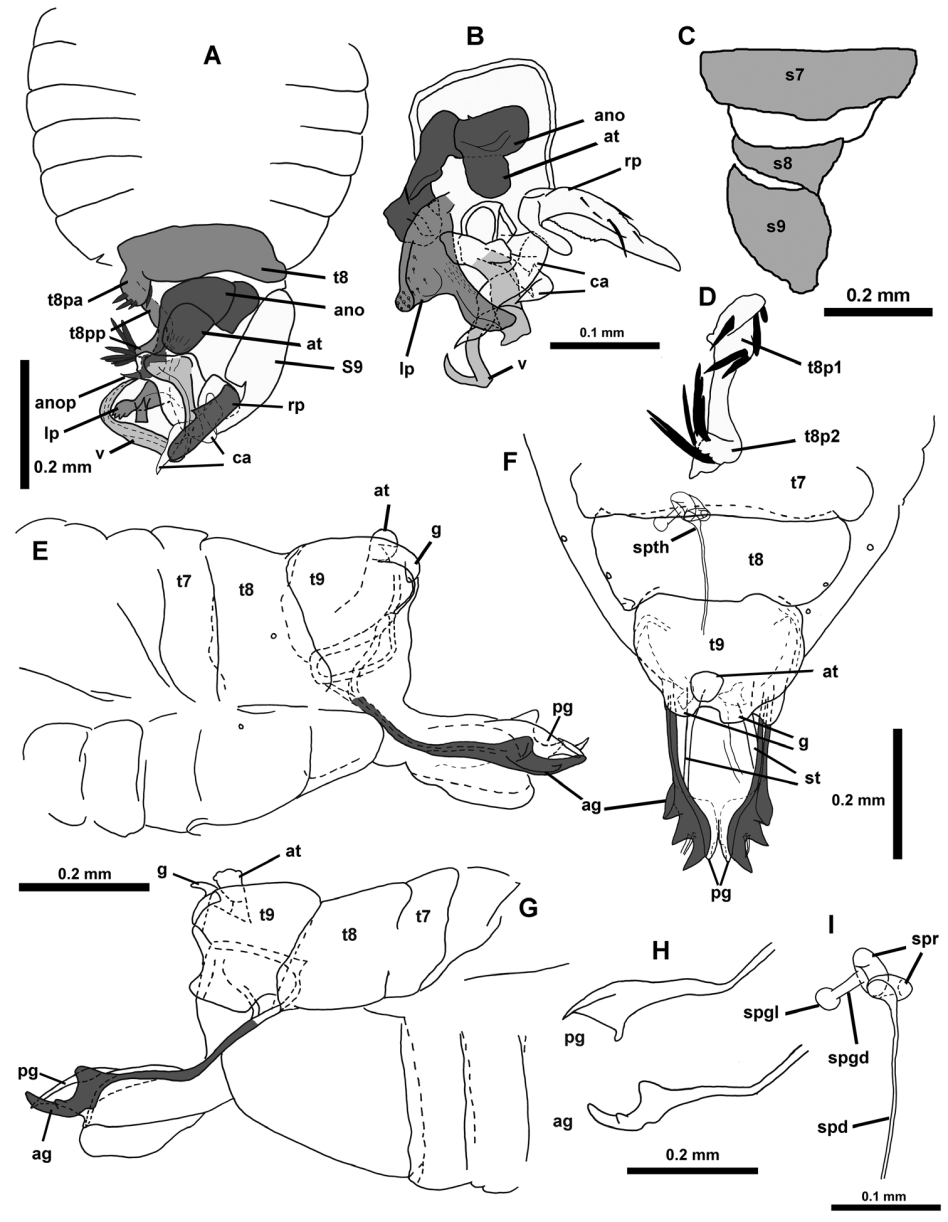
*Hypselosomops*: **A** male abdomen in dorsal view **B** male genitalia in dorsal view **C** male abdominal segments seven, eight, nine in ventral view **D** male tergite eight in lateral view **E** left side of female abdomen in lateral view **F** female abdomen in dorsal view **G** right side of female abdomen in lateral view **H** gonapophysis (pg- posterior gonapophysis, ag- anterior gonopophyses) **I** spermatheca including part of duct.

**Figure 5.**
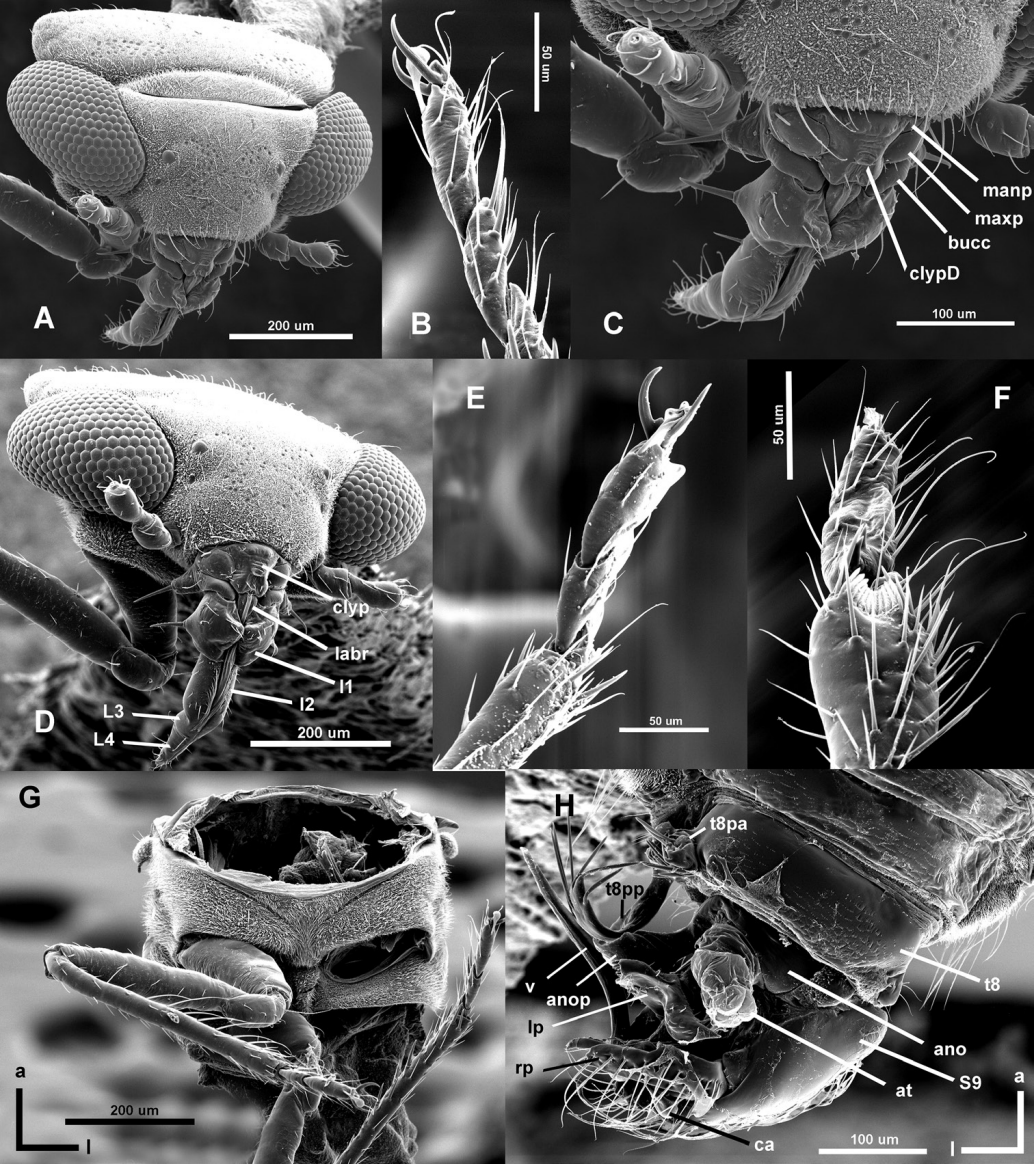
*Hypselosomops* male, SEM images: **A** frontal-lateral-dorsal view of head **B** hind tarsus **C** close-up of frontal mouthparts **D** frontal view of head **E** mid tarsus: **F** fore tarsus **G** ventral view of thorax with head, pronotum and left mid and hind legs removed (showing the peg-like structure between legs hypothesized to be used for jumping) **H** dorsal view of genitalia.

**Figure 6.**
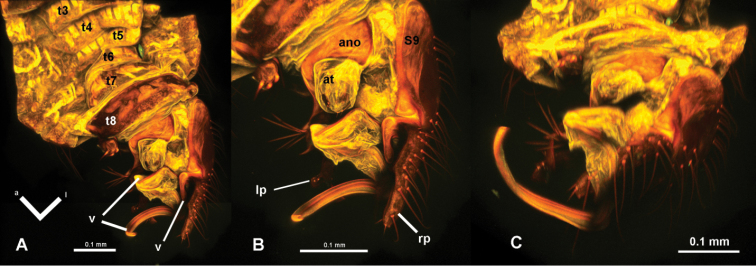
*Hypselosomops* male, confocal images: **A** dorsal view of genitalia **B** dorsal view of genitalia tilted slightly left **C** dorsal view of genitalia tilted slightly more to the left.

**Figure 7.**
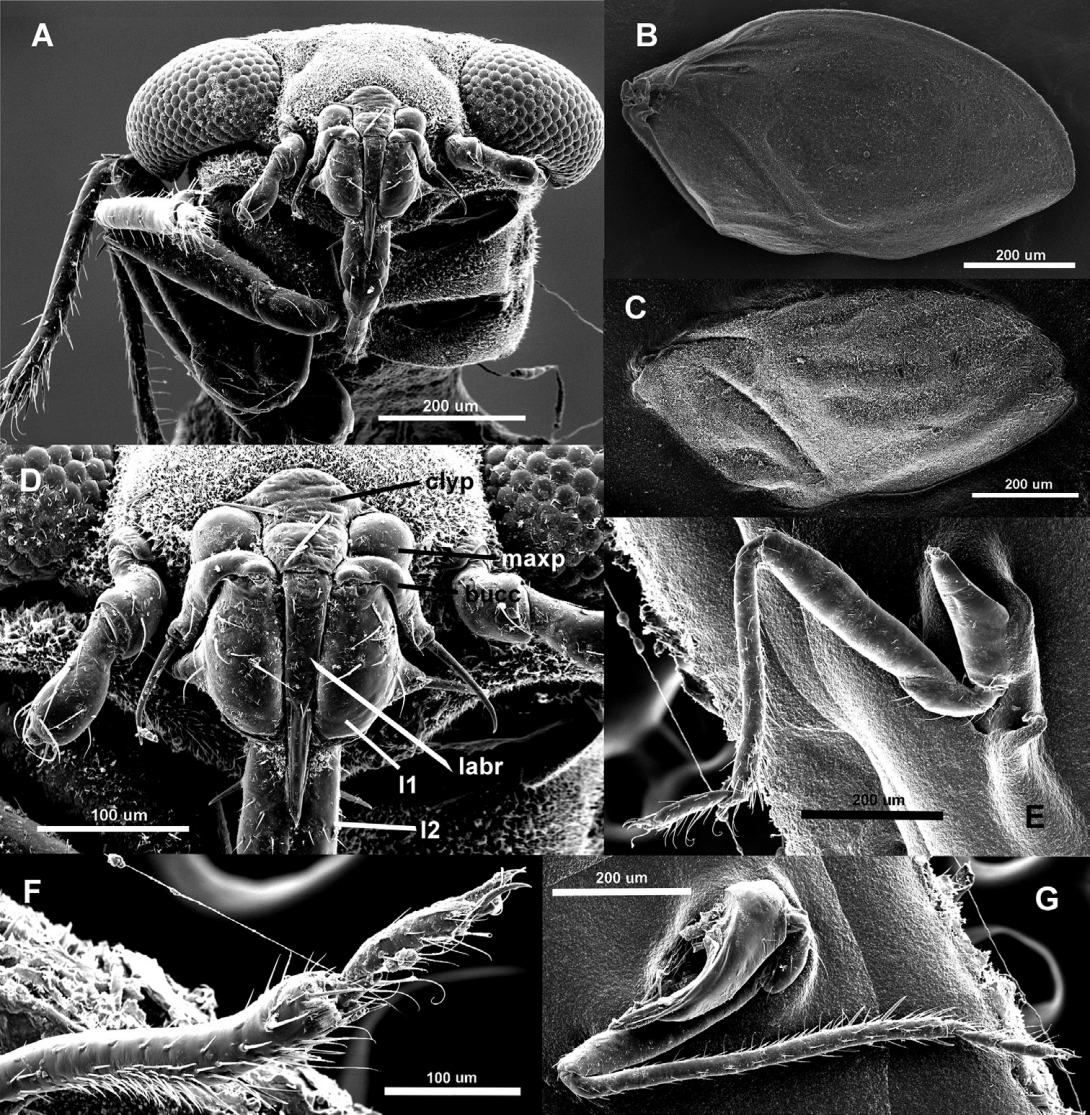
*Hypselosomops* female, SEM images: **A** front-ventral view of head and thorax **B** inner surface of wing **C** outer surface of wing **D** close-up frontal view of mouthparts **E** mid leg **F** front leg **G** hind leg

Figures 2 , 3 , 4 , 5 , 6 , 7

#### Type species.

*Hypselosomops pecki* sp. n.

#### Diagnosis.

Distinguished among Hypselosomatinae by males with two wing morphs (macropterous and submacropterous); slightly enlarged and dorsally expanded first labial segment lacking anterior “teeth-like” setae (Fig. 5D); third and fourth labial segments distinctly separated (Fig. 5D); clypeus without macrosetae but with medial apical indentation with small basal flap and a row of five small setae at apex (Fig. 5C); large seta with strongly expanded apex originating dorsoapically on third tarsal segment of all legs; tergite eight with bipartite process on left side (Fig. 4A, D); two conjunctival appendages at base of vesica (Fig. 4A, B). Most similar among New World Hypselosomatinae to *Ommatides* in coloration (Fig. 12). Among Old World hypselosomatine genera similar to *Duonota* and *Rectilamina* (Hill 1984), with which it shares the absence of clypeal macrosetae in males and the presence of three clypeal macrosetae in females, as well as the modifications of tergite eight in males and the presence and shape of the spermatheca in females. Distinguished from all hypselosomatine genera by the structure of the clypeus and the process of tergite eight.

#### Description.

**Male. Coloration.** Head, thorax, abdomen ventrally, and forewing light to dark brown, with pale transverse band proximally on forewing; legs yellow, coxae brown; abdomen dorsally pale; genital capsule and genitalia light to dark brown. **Surface and Vestiture.** Clypeus without macrosetae, with a depression in apical half and a row of five small setae at apex; buccula and first labial segment each with a pair of stout ventrolateral macrosetae; second labial segment with pair of lateral macrosetae (Fig. 5C); first antennal segment with five setae; ratio of length of third antennal segment and fourth antennal segment approximately 0.93; fore femur without anterior macroseta; hind tibia with four erect medium-length macrosetae ventrally on distal half; second tarsomere of hind leg with one macroseta anteroventrally (Fig. 5B); projections on left side of tergite eight with distinctive spines at apices, anteriormost process of tergite eight with simple spines, posterior process of tergite eight with spines with differentiated bases; anophore without distinctive setae (Fig. 5H). **Structure.** First labial segment slightly enlarged, with small frontally converging lobes (Fig. 5C, D); clypeus with medial apical indentation with small basal flap (Fig. 5C); ratio of pronotal collar length to pronotum length 0.23 (macropterous male), 0.28 (submacropterous male); disc steeply declivous, not decurrent anteriorly; collar slightly depressed below pronotum (Fig. 2); postnotum subrectangular (i.e. the freely projecting portion is straight along most of the posterior margin, curving only laterally, as in *Rectilamina* Hill); ratio of height of fore femora to length of fore femora 0.25 in macropterous and submacropterous morph; tarsal formula 2-3-3; large seta with strongly expanded apex originating dorsoapically on third tarsal segment of all legs; pretarsus with two long, thin parempodia with slightly expanded and flattened apex; inflated arolium absent (Fig. 5B, E, F); ratio of length of hind tibia to width of pronotum 1.00 (macropterous), 1.11 (submacropterous); fore wing polymorphic (macropterous or submacropterous); wing venation as in Fig. 3A, C, D; trapezoidal cell variable; discal cell elongate rectangular; in macropterous form first anal vein not meeting cubitus, in submacropterous form first anal vein meeting cubitus forming cubital cell; macropterous form with full length hind wing, submacropterous form with greatly reduced hind wing; abdomen with two pairs of spiracles (on sternites seven and eight). **Genitalia.** Posterior margin of sternite seven simple, very slightly asymmetrical (Fig. 4C); right side of tergite eight with small projection, left side with two projections (one short and semi-triangular, and one long, slender and curving dorsad), each with distinctive spines on apices (Fig. 4A, D); sternite eight free of sternite seven and with nearly medial triangular lobe (Fig. 4C); anophoric process long, thick and curving dorsally but without articulations (Fig. 4A); right paramere mitten-shaped with short process at base forming a “thumb”; left paramere scapula-shaped with a large thumb-shaped projection (Fig. 4A, B); vesica of average length with two bends, and two conjunctival appendages at base, one long and pointed, the other shorter and pointed and with a 90 degree bend just before the point (Fig. 4A, B).

**Female. Coloration.** Similar to male. **Surface and Vestiture.** First antennal segment with five macrosetae; ratio of third antennal segment and fourth antennal segment approximately 0.93; clypeus not inflated with three (two basal, one at tip) macrosetae; second labial segment with pair of lateral macrosetae, buccula with pair of lateral macrosetae (Fig. 7A, D); fore femur without anterior macroseta; hind tibia with four erect medium-length macrosetae ventrally on distal half; second hind tarsomere with one macroseta anteroventrally (Fig. 7G); sternite with narrow U-shaped shiny area reaching about 1/3 towards anterior margin (Fig. 2). **Structure.** Similar to male, but ratio of pronotal collar length to pronotum length 0.18; disc steeply declivous, not decurrent anteriorly; collar slightly depressed below pronotum (Fig. 2); postnotum short and rectangular; ratio of height of fore femora to length of fore femora 0.29; tarsal formula 2-2-3; pretarsus as in male: ratio of length of hind tibia to width of pronotum 1.03; submacropterous; wing venation as in Fig. 3; discal cell elongate rectangular; first anal vein meeting cubitus to form cubital cell; hind wing lacking; tergite eight without posterolateral projections (Fig. 4E, G); abdomen with two pairs of spiracles (on tergite seven and sternite eight). **Genitalia.** Anterior gonapophysis with three teeth, no subapical serration (Fig. 4E, G, H); posterior gonapophysis with two teeth (Fig. 4E, G, H); median styloid bifurcate (Fig. 4F); gonoplacs round apically (Fig. 4F); spermathecal gland spherical; spermathecal gland duct straight; spermathecal reservoir globular with two bends; spermathecal duct long and straight (Fig. 4I).

**Nymphs**: For habitus and size of first, second, fourth, and fifth instar nymphs, see Fig. 2 and Table 1.

#### Etymology.

“*Hypselosom*” from *Hypselosoma*, the type genus of Hypselosomatinae, and “*ops*” which is Greek for “having the appearance of”, due to the similarity of this genus to *Hypselosoma*. The gender is feminine.

#### Notes.

The medial apical indentation with small basal flap on the clypeus (Fig. 5C) is a unique character, the likes of which have been described in only one other species, *Hypselosoma oncerochilotum* Hill, from Queensland, Australia (Hill 1987). We suspect that this feature may be associated with an organ of unknown function, however, histological studies would be required to confirm this hypothesis. Also the large seta with strongly expanded apex originating dorsoapically on the third tarsal segment of all legs is a distinctive character similar to the larger dorsal seta on the apical tarsomeres in *Silhouettanus* Hill (Hill 2014).

#### Distribution.

Specimens of this genus have been collected in Cuba and the Dominican Republic.

### Hypselosomops pecki sp. n.

Taxon classificationAnimaliaHemipteraSchizopteridae

http://zoobank.org/5C2ABC84-8700-4E5F-BD7A-4DAD3D1E20D1

#### Material.

***Type material*.** **Holotype**, male, slide mounted in Canada balsam: CUBA: Santiago, Gran Piedra, Isabelica, 20.08333°N 75.6°W, 1100 m, 07 Dec 1995 - 17 Dec 1995, S. B. Peck (UCR_ENT 00091116) (FMNH). **Paratypes**: CUBA: Santiago: Gran Piedra Co.: Gran Piedra, Isabelica, 20.08333°N 75.6°W, 1100 m, 06 Dec 1995, S. B. Peck, 2 females (UCR_ENT 00098864, UCR_ENT 00098865) (FMNH); 07 Dec 1995 - 17 Dec 1995, S. B. Peck, 1 female (UCR_ENT 00091115), 1 male slide mounted in Canada balsam (UCR_ENT 00091117), 5 point mounted males (UCR_ENT 00091118, UCR_ENT 00096904–UCR_ENT 00096907) (FMNH); 14 Dec 1995, S. B. Peck, 3 males (UCR_ENT 00096909–UCR_ENT 00096911), 1 female slide mounted in Canada balsam (UCR_ENT 00096912), 3 point mounted females (UCR_ENT 00096913–UCR_ENT 00096915), 7 juveniles (UCR_ENT 00096916-UCR_ENT 00096922) (FMNH). Gran Piedra, Meteo Radar Station, 20.00944°N 75.62722°W, 1100 m, 06 Dec 1995 - 17 Dec 1995, S. B. Peck, 2 males (UCR_ENT 00096908, UCR_ENT 00091120) (FMNH). DOMINICAN REPUBLIC: La Vega: 8 km. S. Constanza on Hwy. 41, 18.83538°N 70.71912°W, 02 Sep 1997, P. W. Kovarik, 2 males (UCR_ENT 00094277, UCR_ENT 00094278) (TAMU).

#### Diagnosis.

As in generic diagnosis.

#### Description.

As in generic description.

#### Measurements.

See Table 1.

#### Etymology.

Named in honor of Stewart B. Peck (Carleton University, Ottawa), who collected most known specimens of this species. A noun in genitive case.

### Hypsohapsis gen. n.

Taxon classificationAnimaliaHemipteraSchizopteridae

http://zoobank.org/659AB66E-984D-40EE-9EAE-1119A9D04CD3

**Figure 8.**
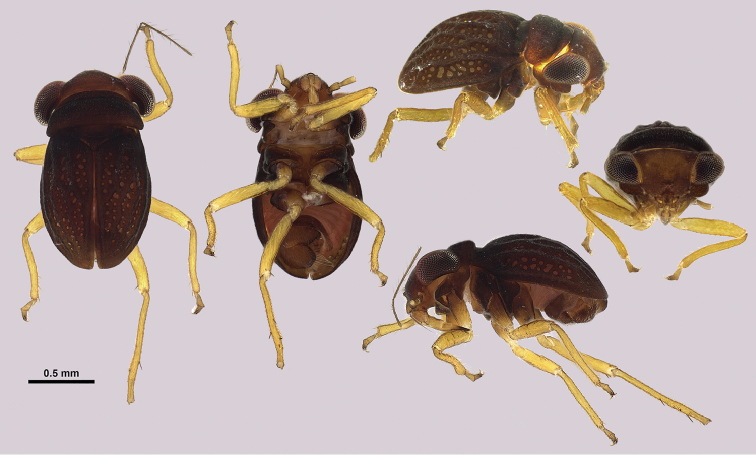
Dorsal, ventral, lateral right, lateral left, and frontal habitus of *Hypsohapsis takiyae*.

**Figure 9.**
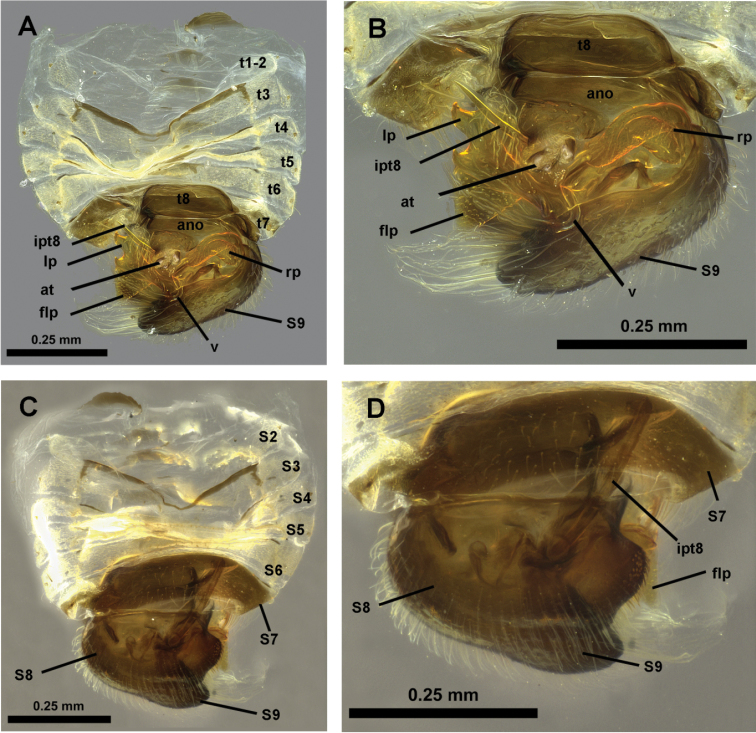
*Hypsohapsis takiyae* **A** dorsal view of abdomen **B** closer view of dorsal abdomen **C** ventral view of abdomen **D** closer view of ventral abdomen

**Figure 10.**
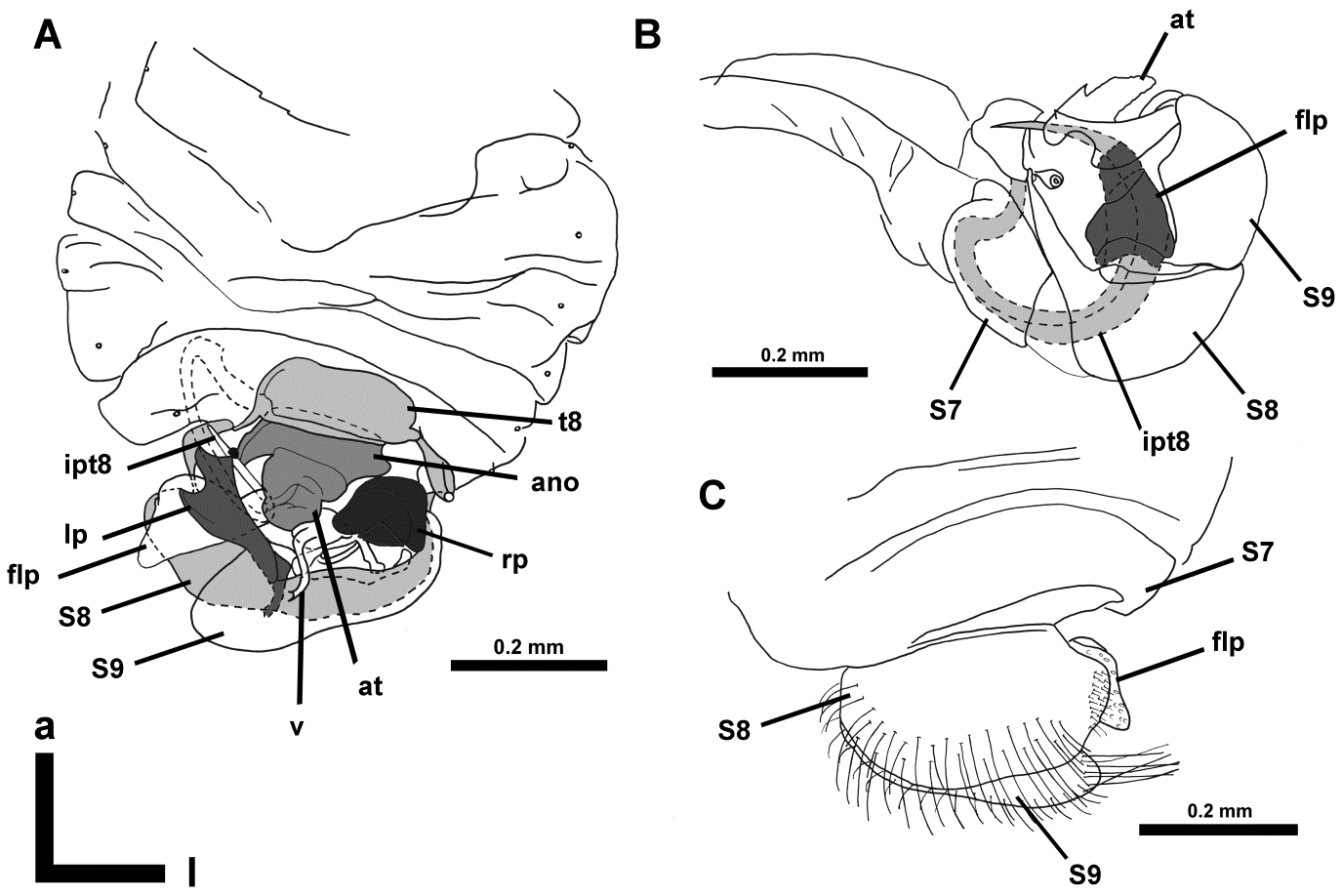
*Hypsohapsis takiyae* **A** dorsal view of abdomen **B** lateral left view of abdomen **C** ventral view of abdomen

**Figure 11.**
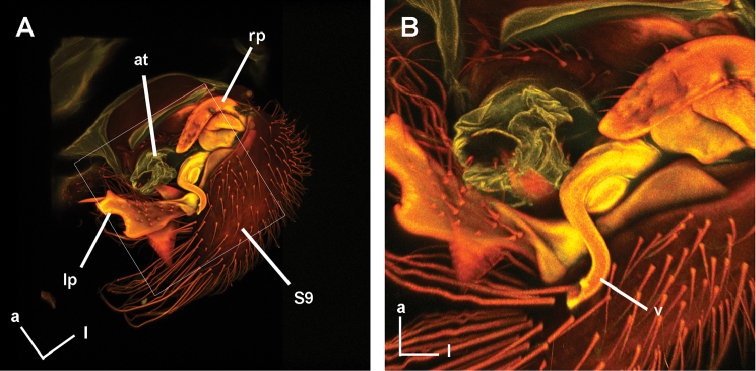
*Hypsohapsis takiyae*, confocal images: **A** dorsal view of abdomen **B** closer view of dorsal abdomen.

Figures 8 , 9 , 10 , 11

#### Type species.

*Hypsohapsis takiyae* sp. n.

#### Diagnosis.

Distinguished among Hypselosomatinae by first labial segment not dorsally expanded; clypeus with five macrosetae; 3 not 1 or 2 pairs of ventrolateral macroseta at base of mouthparts (bucculae and first two labial segments); fore femur without macrosetae; areolate fore wing with only traces of veins (Fig. 8); sternite eight large and capsule like; process of tergite eight looping internally and projecting externally as a long, thin process; a second scale like genitalic process with uncertain origin; sternite nine with a large lobe to the left at end (Figs 9 and 10).

#### Description.

**Male. Coloration.** Head, thorax, and forewing brown (including eyes); legs yellow; basal portion of coxae brown, abdomen pale; genital capsule and genitalia brown. **Surface and Vestiture.** Clypeus with five macrosetae (two basal, three at tip); first labial segment with a pair of stout lateral macrosetae, second labial segment with pair of lateral macrosetae, buccula with pair of lateral macrosetae; fore femora without anterior macroseta; ratio of length of hind tibia to width of pronotum 0.97; hind tibia with five erect macrosetae ventrad on distal half. **Structure.** ratio of pronotal collar length to pronotum length 0.21; disc steeply declivous, not decurrent anteriorly (Fig. 8); collar slightly depressed below pronotum (Fig. 9); postnotum of unknown shape (requires further dissection of specimen); ratio of height of fore femora to length of fore femora 0.30; tarsal formula 2-3-? (third tarsal segment hind of hind legs missing); pretarsus of front and middle legs with inflated arolia (pretarsus of hind leg unknown); elytrous with areolate and irregular coarse sculpturing; abdomen with seven pairs of spiracles (on sternites two through eight). **Genitalia.** Hind margin of sternite seven simple; left side of tergite eight with a process that loops around internally and then projects externally as a long thin process; right side of tergite eight with small projection and large external spiracle; left side with short slender projection that connects to sternite; sternite eight free of sternite seven; sternite eight large and capsule like, with elongated slender left margin that connects to tergite eight left-sided process; a flap like genitalic process (flp) on the left side posteriorly to segment eight is of uncertain origin; anophoric appendage absent; sternite nine lobe shaped to the left (in dorsal perspective) (Figs 9, 10 and 11).

**Female.** Unknown.

#### Etymology.

“*Hypso*” from *Hypselosoma*, the type genus of Hypselosomatinae, and “*hapsis*” which is Greek for “net” in reference to the netlike appearance of the fore wing due to their areolate and irregular, coarse sculpturing. The gender is feminine.

#### Notes.

This currently monotypic genus is described from a single male specimen from southern Brazil. It is clearly set apart from all described genera of Hypselosomatinae by the unique and complicated structure of the male genitalia. In particular, the seemingly internal process of segment eight is unknown among described Dipsocoromorpha. Flap like processes of the genitalia occur in other Schizopteridae, but the origin of this process in *Hypsohapsis* is unclear. Unfortunately, dissection of the abdomen would be necessary for a detailed study of the above mentioned structures. Since only one specimen of the species was collected, we have limited our observations to studying the intact abdomen.

#### Distribution.

One specimen of this genus has been collected in Brazil.

### Hypsohapsis takiyae sp. n.

Taxon classificationAnimaliaHemipteraSchizopteridae

http://zoobank.org/521348ED-75EC-49B0-9E90-A347853B3281

#### Material.

***Type material*.** **Holotype**, male, slide mounted in Canada balsam: BRAZIL: Sao Paulo: Ubatuba, Picinguata, -23.37743,-44.83733, 1997–2001, Nessimain, J.L. & Takiya, D.M. (UCR_ENT 00111639) (UFRJ).

#### Diagnosis.

As in generic diagnosis.

#### Description.

As in generic description.

#### Measurements.

See Table 1.

#### Etymology.

Named in honor of Daniela Takiya who collected this specimen and loaned it to us. A noun in genitive case.

## Discussion

Based on our extensive specimen sorting efforts of Dipsocoromorpha with emphasis on Schizopteridae as part of the NSF-funded ARTS Litter Bug project, it has become clear that Hypselosomatinae are more widely distributed in the New World than previously documented and that a number of undescribed species remain to be described. The present contribution focuses on two new genus-level taxa, but descriptions of additional species are in progress and the analysis of molecular data will provide a phylogenetic framework for both New and Old World Hypselosomatinae (Hoey-Chamberlain and Weirauch, in prep.). The three previously described New World genera share a number of morphological characters. *Ommatides* and *Williamsocoris* both possess dorsally expanded first labial segments, while the male genitalia in *Williamsocoris* and *Glyptocombus* are very similar, both with a fingerlike process apically on sternite nine. The males of both *Ommatides* and *Glyptocombus* are submacropterous and elytrous. We have not been able to recover additional specimens of *Ommatides insignis* beyond the male holotype and certain details of the male genitalia therefore remain unknown. We were also unable to locate specimens that Wygodzinsky (1960) suspected to represent an undescribed species of *Glyptocombus* from southeastern Brazil, but for which he did not indicate the depository. Finally, we have discovered female hypselosomatine specimens that derive from the same collection events as males of *Williamsocoris* and *Glyptocombus* and we suspect that they represent conspecific females. These females lack the dorsally expanded first labial segment making genus-level identification of unassociated females impossible. We suspect that a more thorough study of described and undescribed species of *Williamsocoris* and *Glyptocombus* may lead to the conclusion that the two genera are synonyms. In contrast, the two new genera that we describe here are significantly different from the previously described genera, despite the fact that *Hypselosomops* possesses a slightly expanded first labial segment similar to *Ommatides* and *Williamsocoris*, and *Hypsohapsis*, *Ommatides*, and *Glyptocombus* share the elytrous condition of the hemelytron.

In a series of taxonomic publications focusing on Hypselosomatinae, Hill (1980, 1984, 1985, 1987, 1991, 2013) established a set of characters that are useful for distinguishing genera, including the number of macrosetae on the clypeus, tarsal formula, wing venation and number of marginal cells beyond the costal cell, and the presence/absence or degree of sclerotization and shape of the spermatheca. In the following, we discuss some of these diagnostic features in the context of the two newly discovered and described New World genera. *Hypsohapsis* shares the presence of five macrosetae on the clypeus with *Hypselosoma*, while females of *Hypselosomops* share three macrosetae with the previously described New World genera, the fossil taxa, and four of the Old World genera (*Pateena*, *Duonota*, *Ordirete*, and *Macromannus*) and males lack clypeal macrosetae similar to the Old World genus *Rectilamina*. Among New World genera, the tarsal formula of the males of *Glyptocombus* and *Williamsocoris* is 3-3-3, but it is 2-2-3 in the female of *Glyptocombus*; in contrast, the male of *Hypselosomps* has a tarsal formula of 2-3-3, while the formula of the female is 2-2-3. The tarsal formula of *Ommatides* is unknown (most legs and all tarsi missing in the holotype and single known specimen). Sexual dimorphism in the tarsal formula is fairly common across Schizopteridae (Emsley 1969).

Even though a diagnostic feature of Hypselosomatinae, the default number of four marginal cells can be modified, especially in micropterous and elytrous taxa. Four marginal cells are clearly visible in most macropterous taxa such as *Williamsocoris* and the male, macropterous form of *Hypselosomops*, but *Glyptocombus*, *Ommatides*, and *Hypsohapsis* have reticulate wings and/or reduced wing venation. Wing type in Hypselosomatinae can vary within species, but even within the same sex of the same species. Sexual dimorphism with respect to wing type occurs in taxa in both the Old (e.g., *Rectilamina*, *Duonota*, and *Hypselosoma*) and New Worlds (undescribed species of *Williamsocoris* and *Glyptocombus*; pers. obs.). Only *Hypselosomops pecki* and *Pateena elimata* Hill (Hill 1980) are currently known to comprise both micropterous and submacropterous wing types in the male.

**Figure 12.**
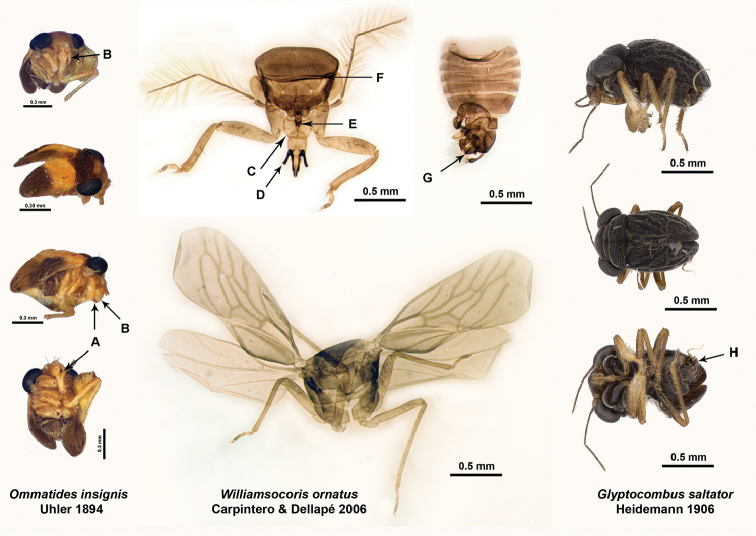
*Ommatides insignis* holotype: **A** enlarged first labial segment **B** anterior teeth like setae; *Williamsocoris ornatus* holotype: **C** enlarged first labial segment **D** horn like or tubular structure occupying third and fourth labial segments **E** anterior teeth like setae of first labial segment **F** pronotal collar **G** projection of genital capsule; and *Glyptocombus saltator* non-type: **H** projection of genital capsule

Carpintero and Dellapé (2006) reported the pronotal collar to be absent in *Williamsocoris*. We argue that the structure indicated by arrow F in Figure 12 represents the collar; a collar is also present in specimens that we have discovered through our sorting efforts and that likely represent undescribed species of *Williamsocoris*. Hill (2013) mentions that in the Old World genus *Cryptomannus* the pronotal disc overlaps the collar. A similar situation in *Williamsocoris* before clearing and slide mounting could have resulted in this misinterpretation.

Although considered to be of value as a diagnostic feature at the genus level, comparative documentation of the spermatheca across Hypselosomatinae is rather incomplete. Females of *Williamsocoris*, *Glyptocombus*, *Ommatides*, and *Hypsohapsis* remain unknown and the spermatheca is assumed to be absent or poorly sclerotized in *Hypselosoma* (Hill 1980, 2013). In the few taxa for which the spermatheca has been documented, namely *Rectilamina* (Hill 1980), *Duonota* (Hill 1984) and *Hypselosomops* it is well-sclerotized and pigmented, with a well-defined spermathecal gland, gland duct, reservoir, and duct.

The number of abdominal spiracles can vary dramatically between hypselosomatine genera, although variation can also occur within a genus. Hill (2013) confirmed three abdominal spiracles in species of *Hypselosoma* from Australia, New Zealand and New Caledonia, while Esaki and Miyamoto (1959) reported that the first five spiracles are reduced in *Hypselosoma hirashimai* Esaki & Miyamoto from Japan, resulting in only three pairs of spiracles. Species of *Pateena* can have five or six (Hill 2013) or even seven abdominal spiracles (on segments two through eight) (Hill 1980). Hill (1980) found six spiracles (on segments two through seven) on a specimen of *Glyptocombus* sp. that Allen and Carlton (1989) later suspected to be *Glyptocombus saltator* Heidemann. *Hypselosomops* has two pairs of abdominal spiracles, *Hypsohapsis* seven pairs, but the condition remains unknown in *Williamsocoris*. Given that spiracle numbers and arrangement are variable within a genus, this character should probably not used as a diagnostic feature at the genus level unless studied in a broader sample of species.

Phylogenetic hypotheses are currently unavailable for Hypselosomatinae and our understanding of character evolution in the group is therefore limited to speculations. *Hypsohapsis* shares a number of morphological features with *Macromannus* and *Ordirete* including the three clypeal macrosetae, elytrous wings with reticulate/areolate pattern and the simple sternite nine with a rounded lobe. *Hypselosomops* in contrast more closely resembles *Duonota* and *Rectilamina* due to the lack of clypeal macrosetae on males and the three clypeal macrosetae on females, as well as, the modifications of tergite eight in males and the presence and shape of the spermatheca in females. Given some of the extraordinary morphologies of Hypselosomatinae, the worldwide distribution with small endemic species ranges, and the fossil record that dates back to more than 100 my (Perrichot et al. 2007, Azar and Nel 2010), a phylogenetic hypothesis for the group is long overdue and will also allow for ancestral state reconstructions of morphological characters that will improve our understanding of the evolutionary history of this charismatic taxon.

## Supplementary Material

XML Treatment for Hypselosomops

XML Treatment for Hypselosomops pecki

XML Treatment for Hypsohapsis

XML Treatment for Hypsohapsis takiyae
